# A Role for *Drosophila* Amyloid Precursor Protein in Retrograde Trafficking of L1-Type Cell Adhesion Molecule Neuroglian

**DOI:** 10.3389/fncel.2019.00322

**Published:** 2019-07-12

**Authors:** Tyrone Penserga, Sirisha Rani Kudumala, Richelle Poulos, Tanja Angela Godenschwege

**Affiliations:** Department of Biological Sciences, Florida Atlantic University, Jupiter, FL, United States

**Keywords:** amyloid precursor protein, axonal transport, neuroglian, *Drosophila*, retrograde trafficking

## Abstract

The role of the Amyloid Precursor Protein (APP) in the pathology of Alzheimer’s disease (AD) has been well studied. However, the normal function of APP in the nervous system is poorly understood. Here, we characterized the role of the *Drosophila* homolog (APPL) in the adult giant fiber (GF) neurons. We find that endogenous APPL is transported from the synapse to the soma in the adult. Live-imaging revealed that retrograde moving APPL vesicles co-traffic with L1-type cell adhesion molecule Neuroglian (Nrg). In APPL null mutants, stationary Nrg vesicles were increased along the axon, and the number of Nrg vesicles moving in retrograde but not anterograde direction was reduced. In contrast, trafficking of endo-lysosomal vesicles, which did not co-localize with APPL in GF axons, was not affected. This suggests that APPL loss of function does not generally disrupt axonal transport but that APPL has a selective role in the effectiveness of retrograde transport of proteins it co-traffics with. While the GF terminals of APPL loss of function animals exhibited pruning defects, APPL gain of function had no disruptive effect on GF morphology and function, or on retrograde axonal transport of Nrg. However, cell-autonomous developmental expression of a secretion-deficient form of APPL (APPL-SD), lacking the α-, β-, and, γ-secretase cleavage sites, resulted in progressive retraction of the GF terminals. Conditional expression of APPL-SD in mature GFs caused accumulation of Nrg in normal sized synaptic terminals, which was associated with severely reduced retrograde flux of Nrg labeled vesicles in the axons. Albeit β-secretase null mutants developed GF terminals they also exhibited Nrg accumulations. This suggests that cleavage defective APPL has a toxic effect on retrograde trafficking and that β-secretase cleavage has a function in Nrg sorting in endosomal compartments at the synapse. In summary, our results suggest a role for APPL and its proteolytic cleavage sites in retrograde trafficking, thus our findings are of relevance to the understanding of the endogenous role of APP as well as to the development of therapeutic treatments of Alzheimer’s disease.

## Introduction

Amyloid Precursor Protein (APP) as well as APP-like protein 1 and 2 (APLP1 and APLP2) are a highly conserved family of type 1 transmembrane proteins. Human APP and its proteolytic cleavage has been extensively studied due to its relevance in Alzheimer’s disease (AD). APP can either be cleaved by α-secretase at the plasma membrane (non-amyloidogenic pathway) or by β-site APP-cleaving enzyme 1 (BACE-1) in early endosomes (amyloidogenic pathway) resulting in soluble extracellular sAPPα and sAPPβ fragments as well as C83 and C99 transmembrane fragments, respectively (Sisodia, 1992; Rajendran et al., 2006; Sannerud et al., 2011; Rajendran and Annaert, 2012). Subsequent cleavage of C99 by γ-secretase/presenilin in the late endolysosomal compartments generates a cytoplasmic C-terminal fragment as well as small Aβ40 or Aβ42 peptides, which are the primary constituent of amyloid plaques, the pathological hallmark of AD (Masters et al., 1985; Sisodia, 1992; Takahashi et al., 2002; Vieira et al., 2010; Glenner and Wong, 2012; Rajendran and Annaert, 2012; van der Kant and Goldstein, 2015). The toxic effects of Aβ42 oligomers on receptor signaling and numerous other cellular processes such as axonal transport have been extensively studied (Sakono and Zako, 2010; Puzzo and Arancio, 2013; Wang et al., 2015). However, little is known about the physiological role of APP proteins, which is in part due to redundant functions of APP, APLP1, and APLP2 and that triple knockout mice are lethal (Herms et al., 2004; Wang et al., 2005; Priller et al., 2006; van der Kant and Goldstein, 2015). Here, we further characterized the function of the sole *Drosophila* homolog of APP, the β-amyloid protein precursor-like (APPL), in an adult central nervous system neuron.

Proteolytic cleavage of APPL by α-, β-, and, γ-secretase is conserved, and although the sequence of Aβ42 is not conserved, APPL does produce an Aβ-like fragment that has neurotoxic effects (Carmine-Simmen et al., 2009; Poeck et al., 2012; Cassar and Kretzschmar, 2016). Null mutants (*APPL^d^*) are viable, exhibit learning, and other behavioral defects (Luo et al., 1990, 1992). Loss of function phenotypes described for photoreceptor cells and in the mushroom bodies imply a role for APPL in neuronal outgrowth (Singh and Mlodzik, 2012; Mora et al., 2013; Soldano et al., 2013; Singh et al., 2017). At the larval neuromuscular junction, the bouton numbers are reduced in *APPL^d^* mutants, while APPL gain of function leads to additional boutons revealing that APPL also has function in synaptogenesis (Torroja et al., 1996, 1999a,b; Ashley et al., 2005). In both, APPL gain and loss of function animals, accumulation of vesicles containing synaptotagmin (Syt), or cysteine string protein (Csp) are observed in larval motoneuron axons suggesting a role in axonal transport (Torroja et al., 1999a; Gunawardena and Goldstein, 2001; Gunawardena et al., 2013). Previously, it has been considered that APP or APPL gain of function disrupts axonal trafficking by competing for JIP1/kinesins, but a more recent study shows that it leads to increased calcineurin and GSK-3β signaling via an unknown mechanism, which affects kinesin interaction with synaptotagmin (Gunawardena and Goldstein, 2001; Shaw and Chang, 2013; van der Kant and Goldstein, 2015).

Recently, it has been shown that secreted APP acts as a ligand of GABA_B_R1 to modulate synaptic transmission, while full-length APP forms a complex with GABA_B_R1 to promote its transport to the synapse (Dinamarca et al., 2019; Rice et al., 2019). Vertebrate APP interacts with cellular motor proteins. Although a direct interaction of APP with kinesin is controversial, there are numerous studies that support an indirect interaction of the intracellular domain of APP with kinesins via Fe65 and JNK kinase interacting protein 1 (JIP1) to promote its own anterograde transport (Sabo et al., 1999; Kamal et al., 2001; Matsuda et al., 2001; Scheinfeld et al., 2002; Lazarov et al., 2005; Muresan and Muresan, 2005, 2012; Chiba et al., 2014). This implies, albeit not directly shown, that APP loss of function may affect anterograde transport of proteins that traffic in APP vesicles. However, APP localizes to vesicles that contain both kinesins and dynein, while JIP1 was shown to regulate anterograde as well as retrograde transport of APP via its differential interaction with kinesin and dynein (Szpankowski et al., 2012; Fu and Holzbaur, 2013). In addition, presenilin levels affect APP velocities in antero- and retrograde direction (Gunawardena et al., 2013). However, the same study showed that the velocity of synaptotagmin vesicles is not affected by presenilin, albeit synaptotagmin does accumulate in the axons that lack APPL (Torroja et al., 1999a; Gunawardena and Goldstein, 2001; Gunawardena et al., 2013).

Despite these and numerous other studies, the functional role of APP during axonal transport is not fully understood. It is unclear if APP binding to JIP1 only promotes its own anterograde transport or if it also has a role during retrograde transport, and if APP loss of function selectively affects vesicles it normally traffics in or leads to general axonal transport defects.

**FIGURE 1:**
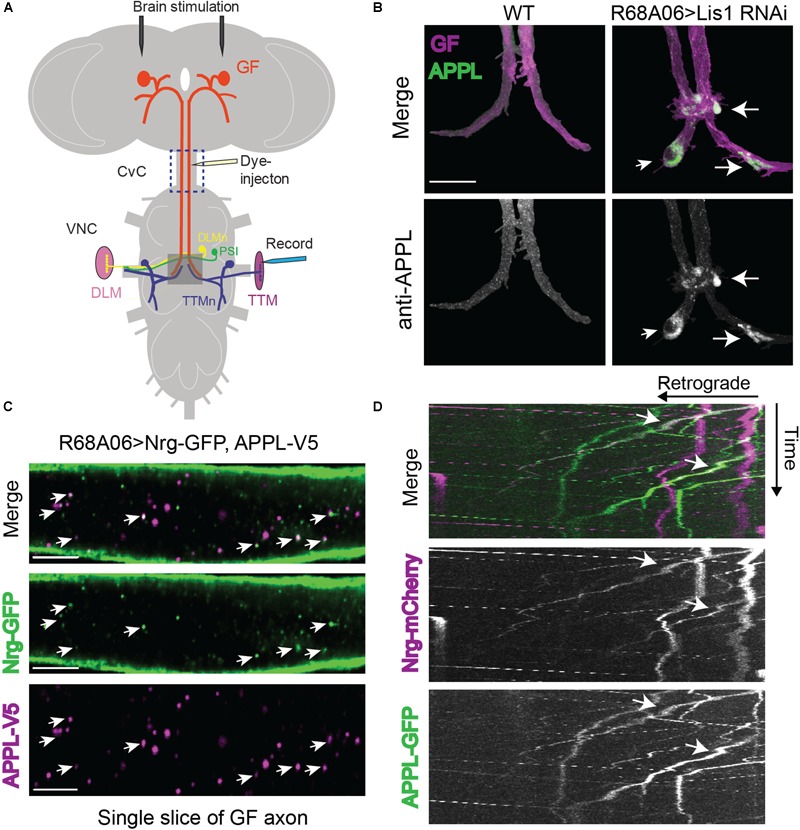
Drosophila Amyloid Precursor Protein (APP) is retrogradely transported and co-traffics with Neuroglian. **(A)** Diagram of Giant Fiber (GF) circuit in the Drosophila nervous system. The GFs synapse with the TTMn and the peripherally synapsing interneurons (PSI) in the ventral nerve cord (VNC). The circuitry output is via the innervation of the jump (TTM) and flight (DLM, dorsal longitudinal muscle) muscles by the TTMn and DLMn (dorsal longitudinal) motoneurons. Live-imaging of GF axons were performed in the CvC (blue square). Sites of dye injections for GF anatomy visualization and placement of stimulation and recording electrodes for electrophysiological analysis are indicated. Gray box shows area of morphological analysis of GF terminals by confocal microscopy in the ventral nerve cord (VNC). **(B)** Immunostaining of endogenous APPL (green top panel and white bottom panel) with an antibody against the N-terminal domain in wild type GFs and in GFs in which Lis1 was cell-autonomously knocked down. GFs (magenta, top panel) were labeled by dye injections of rhodamine-dextran into the GF axons. For better visualization, the GFs were digitally traced and 3D-reconstructed to extract anti-APPL labeling that localizes to the reconstructed GFs (bottom panel). APPL accumulation at GF-TTMn and GF-PSI contact sites in Lis1 knock down animals are indicated by arrows. Scale bar represents 30 μm. **(C)** Immunolabeling of GFP- tagged Nrg (green) and V5-tagged APPL (magenta) in adult GF axons co-expressed using the R68A06 Gal4-driver. Single confocal slices of the different channels in the same plane are shown separately and together to visualize co-localization (arrows). Scale bar represents 5 μm. **(D)** Live-imaging of co-expressed mCherry-tagged Nrg and GFP-tagged APPL in the GF axons (Supplementary Video S1). A small section of the GF axons was photobleached prior to acquisition to reduce background from Nrg labeling at the axonal membrane. Video was obtained at 1 frame per second. Kymographs of APPL-GFP and Nrg-mCherry alone as well as together are shown. Overlapping trajectory of APPL-GFP and Nrg-mCherry vesicles in retrograde direction are indicated by white arrows.

Studies in *Drosophila* larval motoneurons have made significant contributions on the role of APPL in axonal transport. Here, we aim to tease apart the role of APPL in retrograde transport employing our novel giant fiber (GF) neuron model for live imaging of axonal transport in the adult nervous (Kudumala et al., 2017). The GFs are two adult central nervous system neurons that synapse with the Peripheral Synapsing Interneurons (PSI) and the Tergo-Trochanteral Motoneurons (TTMn) to mediate the escape response in the fly (GFs, Figure 1A; Allen et al., 2006). Due to its large axon (diameter 6–8 μm) it provides outstanding resolution for live imaging of axonal trafficking at the single cell level. Using the GF as a model, this study focuses on determining whether APPL has a role in retrograde transport.

## Results

### Retrograde Co-trafficking of APPL With Neuroglian

To determine if endogenous APPL is present and transported from mature GF terminals to the soma we conditionally inhibited retrograde transport by cell-autonomous RNAi knockdown of Lissencephaly-1 (Lis1), a regulatory protein of the dynein motor using the R68A06 or GF-Split Gal4-lines (Vallee and Tsai, 2006). Both Gal4-lines turn on expression only after the GF terminals have formed (Pfeiffer et al., 2008; von Reyn et al., 2014; Kudumala et al., 2017). Immunostaining with APPL antibodies against the extracellular domain showed that APPL accumulates in all GF terminals (*n* = 8) of Lis-RNAi animals but not in wildtype control animals (*n* = 8, Figure 1B). This suggests that vesicles designated for retrograde transport contain either full-length or BACE-cleaved APPL.

In order to assess APPL’s role in retrograde transport, it requires the identification of a protein that co-traffics with APPL in a retrograde manner. We previously showed that L1-type cell adhesion molecule Neuroglian (Nrg) is transported from GF terminals in retrograde direction (Kudumala et al., 2017), while vertebrate homologs of Nrg have been shown to physically interact with APP and co-immunoisolate with APP vesicles (Osterfield et al., 2008; Almenar-Queralt et al., 2014). Therefore, we determined if APPL and Nrg do co-localize in GF axons. Analysis of GF axons expressing V5-tagged APPL and GFP-tagged Nrg revealed that 39.4 ± 4.5% (*N* = 8) of all APPL vesicles co-localized with Nrg (Figure 1D). Similarly, not all Nrg-GFP (34.7 ± 3.2%, *N* = 8) vesicles co-localized with APPL-V5 (Figure 1C). We previously showed that Nrg traffics in fast, continually moving antero- and retrograde vesicles, as well as in slow moving retrograde vesicles that frequently change their velocity during runs (Kudumala et al., 2017). To determine the directionality of APPL/Nrg containing vesicles, we live-imaged trafficking of GFP-tagged APPL and mCherry-tagged Nrg in GF axons in the cervical connective (Figure 1A,D). We found that APPL and Nrg co-traffic in slow moving retrograde but not in anterograde vesicles (Figure 1D and Supplementary Video S1).

### APPL Loss of Function Disrupts Retrograde Transport of Nrg

**FIGURE 2:**
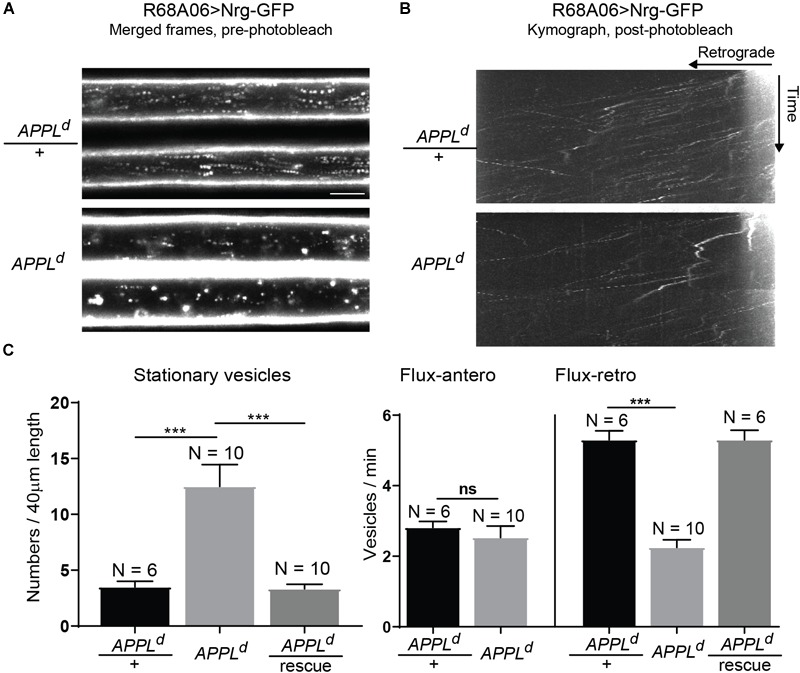
Neuroglian axonal trafficking in APPL loss of function mutants. **(A)** Merged frames of live recordings without photobleaching (Supplementary Videos S2, S3). Moving vesicles produce a “motion-streak,” which is more abundantly seen in wildtype animals than in *APPL*^d^ null mutants. Videos were obtained at one frame per second. Scale bar represents 5 μm. **(B)** Kymographs of axonal transport of Nrg-GFP vesicles in control animals (*APPL*^d^/+) and *APPL*^d^ mutants (Supplementary Videos S4, S5). A small section of the GF axon was photobleached to reduce background from Nrg labeling at the axonal membrane, allowing the visualization of vesicles that enter the bleached region. Videos were obtained at one frame per second. **(C)** Quantification of Nrg-GFP vesicle trafficking in wildtype controls (*APPL*^d^/+), *APPL*^d^ as well as in *APPL*^d^ rescue animals (UAS-Nrg-GFP co-expressed with UAS-APPL in *APPL*^d^ background using R68A06 Gal4-line). Numbers of stationary vesicles, and numbers of vesicles moving in anterograde and retrograde direction (Flux) were analyzed. N indicates numbers of axons assessed. Error bars represents standard error mean. Statistical significance between genotypes was assessed using Student’s *t*-test (^∗^*p* ≤ 0.05, ^∗∗^*p* < 0.01, ^∗∗∗^*p* < 0.001, ns – non-significant, *p* > 0.05).

**FIGURE 3:**
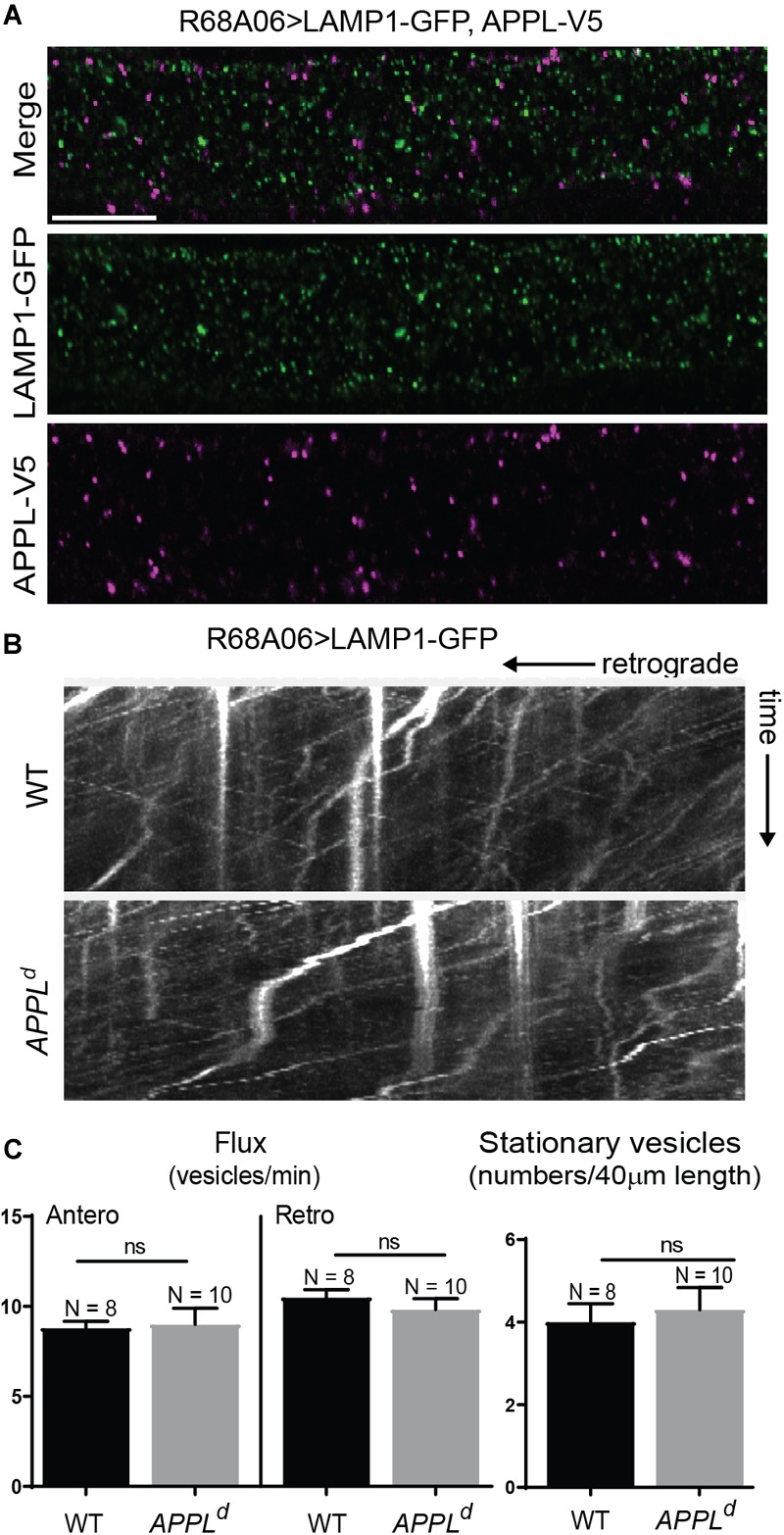
LAMP1 axonal trafficking in APPL loss of function mutants. **(A)** Immunolabeling of GFP- tagged LAMP1 (green) and V5-tagged APPL (magenta) in adult GF axons co-expressed using the R68A06 Gal4-driver. Single confocal slices of the different channels in the same plane are shown separately and together. No co-localization was observed. Scale bar represents 10 μm. **(B)** Kymographs (unbleached) of axonal transport of LAMP1-GFP vesicles in wildtype (WT) control animals and *APPL*^d^ null mutants (Supplementary Videos S6, S7). Videos were obtained at two frames per second. **(C)** Quantification of LAMP1-GFP vesicle trafficking in wildtype and *APPL*^d^ mutants. Numbers of stationary vesicles, and numbers of vesicles moving in anterograde and retrograde direction (Flux) were analyzed. N indicates numbers of axons assessed. Error bars represents standard error mean. Statistical significance between genotypes was assessed using Student’s *t*-test (ns, non-significant, *p* > 0.05).

To determine if APPL loss of function affects axonal transport of Nrg, we expressed Nrg-GFP with the R68A06 Gal4-line in GFs of *APPL^d^* null mutants and live imaged axons of 1–5-day old animals. *APPL^d^* axons contained a significantly higher amount of stationary Nrg vesicles than in *APPL^d^* heterozygote control animals (Figure 2A,C and Supplementary Videos S2, S3), while the flux of Nrg vesicles moving in retrograde but not in anterograde direction was significantly reduced (Figure 2B,C and Supplementary Videos S4, S5). Co-expression of APPL with Nrg-GFP driven by the R68A06 Gal4-line rescued this phenotype; both numbers of stationary vesicles and retrograde flux were similar to control animals (Figure 2C). This demonstrates the cell-autonomous specificity of the observed Nrg trafficking defects in *APPL^d^* null mutants. To further address if APPL loss of function has a general disruptive effect on axonal transport or a more direct role in retrograde transport of Nrg, we assessed trafficking of vesicles that do not co-traffic with APPL. In contrast to Nrg, GFP fused to the cytoplasmic tail of Lysosomal-associated membrane protein 1 (LAMP1) did not co-localize with V5-tagged APPL in GF axons (Figure 3A) but abundantly traffics in antero- and retrograde direction. Analysis of LAMP1-GFP trafficking in GF axons of 1–5-day old *APPL^d^* mutants did not uncover any trafficking defects with respect to numbers of stationary, retrograde or anterograde moving vesicles (Figure 3B,C and Supplementary Videos S6, S7). Together with the above described finding that APPL co-traffics with Nrg in a retrograde manner, these results strongly suggest that APPL has a conserved, direct function in the efficacy of retrograde axonal transport of Nrg vesicles and does not affect transport of vesicles it does not normally traffic with.

### Distinctive Effects of APPL Gain and Loss of Function on GF Morphology and Function

**FIGURE 4:**
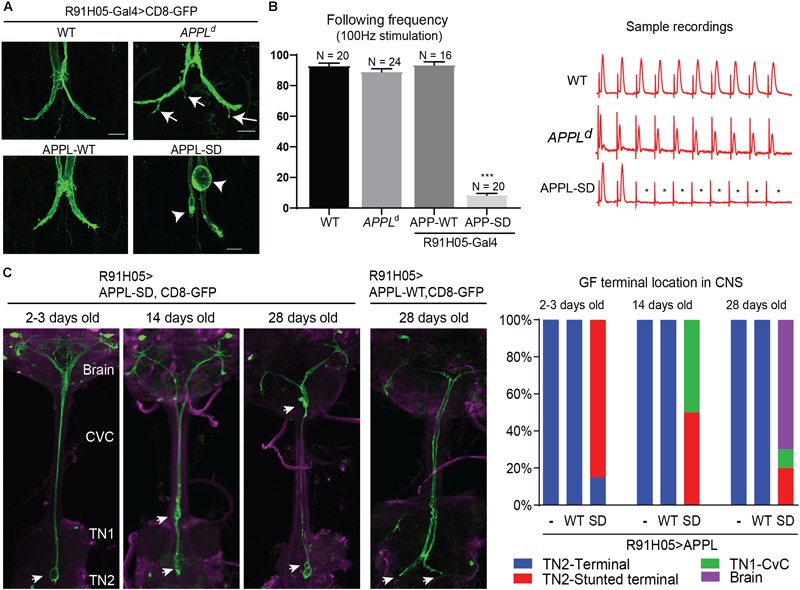
Morphological GF terminal phenotypes in APPL gain and loss of function mutants. **(A)** GF terminal morphology of controls (WT), *APPL*^d^ null mutants and animals overexpressing wildtype (APPL-WT) and secretion-deficient (APPL-SD) APPL throughout GF development. Terminal pruning defects are labeled by arrows. Stunted terminals or GFs with retraction bulbs are labeled by arrowheads. Scale bars represent 20 μm. **(B)** Functional defects of controls (WT), *APPL*^d^ null mutants and animals overexpressing wildtype (APPL-WT) and secretive defective (APPL-SD) APP with the R91H05 Gal4-line. The function of GF to TTMn synapse was determined by quantifying the average number of responses from 10 trains of 10 GF stimulations given at 100 Hz. Quantification is shown in the graph on the left. N indicates numbers of GF-TTMn connections assessed per genotype. Error bars represents standard error mean. Statistical significance between genotypes was assessed using Student’s *t*-test (^∗∗∗^*p* < 0.001). Samples traces of electrophysiological recordings of one train are shown on the right. ^∗^ mark absent responses. **(C)** GF terminal localization in young and aged animals overexpressing wildtype (APPL-WT) and secretion-deficient (APPL-SD) APP. GFs were labeled by co-expression of CD8-GFP. In the left panels the GF terminal localization in the target area (TN2, second thoracic neuromere), first thoracic neuromere (TN1), cervical connective (CvC) or the brain are indicated by arrows. Quantification of GF terminal localization in young and aged animals are shown in the right graphs. Ten or more GFs were assessed for each genotype and time point.

While previously described *APPL^d^* null mutant phenotypes suggest a role for APPL in axon growth and synaptogenesis (Torroja et al., 1999b; Mora et al., 2013; Soldano et al., 2013), interaction of vertebrate APP with the Death Receptor 6 has been suggested to cell-autonomously also regulate axonal pruning (Nikolaev et al., 2009; Kallop et al., 2014; Olsen et al., 2014; Marik et al., 2016). Our finding that APPL is retrogradely transported, implies a role for APPL at the GF terminals. Therefore, we analyzed the morphology and function of GF terminals in *APPL^d^* null mutants by the expression of CD8-GFP membrane marker and with electrophysiological recordings from the TTM (Allen and Godenschwege, 2010). We found that 78% (*n* = 18) of the GF terminals in 1–5-day old *APPL^d^* null mutants exhibited pruning defects (Figure 4A), while the function of the GF synapse was normal (Figure 4B) when compared to control animals. This suggest distinctive functional roles for APPL in different types of neurons, which is consistent with previous studies showing that APPL trafficking and processing is different in different types of neurons (Torroja et al., 1996; Ramaker et al., 2016). Therefore, we determined if the APPL gain of function effects on GF development and function are distinctive from phenotypes observed in the peripheral nervous system.

At the neuromuscular junction overexpression of wild type APPL (APP-WT) and a secretion-defective form of APPL (APPL-SD), lacking the α-, β-, and, γ-secretase cleavage sites, resulted in similar phenotypes, such as the formation of additional boutons (Luo et al., 1990, 1992; Torroja et al., 1999b). Here, we cell-autonomously co-expressed the same constructs with the CD8-GFP throughout GF development using the R91H05 Gal4-line (Pfeiffer et al., 2008; Lee and Godenschwege, 2015). No apparent effects on morphology or function of the GF terminals were observed in animals overexpressing APPL-WT (Figure 4A,B). In contrast, overexpression of mutant APPL-SD protein in 1–5 days old wildtype animals resulted in GFs that were present in the synaptic target region without any pruning defects, but most terminals were stunted or exhibited bulb-like endings (Figure 4A,C). This was associated with functional defects. Electrophysiological recordings from the GF circuit showed that the synaptic connection between the GF and the TTMn was weakened or absent in all animals assessed (Figure 4B). To determine if the phenotype of APPL-SD is progressive, we assessed the GFs in aged animals as well. We found that the numbers of GF terminals that are outside the synaptic contact area in the second thoracic neuromere were increased in 14-day- and 28-day old animals, when APPL-SD but not when APP-WT was expressed and instead the GF endings were seen in the first thoracic neuromere, CvC, or brain (Figure 4C). This suggests that APPL-SD but APP-WT leads to progressive axonal retraction of GF terminals.

### Cleavage Defective APPL Disrupts Retrograde Transport at Synaptic Terminals

We and others previously showed that inhibition of retrograde transport during development results in GF terminal retraction and that when inhibited in mature terminals leads to accumulation of retrogradely transported proteins in GF terminals, such as Nrg (Allen et al., 1999; Kudumala et al., 2017). This suggests that expression of APPL-SD may disrupt retrograde transport of proteins required for GF terminal development and maintenance at the synapse. To further assess this possibility, we determined the localization of endogenous and GFP-tagged Nrg in GF terminals of animals that conditionally expressed APPL-WT or APPL-SD after GF terminal formation with the R68A06 or the GF-Split Gal4-lines. This allows to determine the effects of APPL constructs on trafficking in normal sized terminals without any developmental defects. In addition, we assessed Nrg-GFP localization in terminals of *APPL^d^* null mutants, and determined if expression of APPL-WT and APPL-SD have similar or distinctive effects on axonal transport of Nrg-GFP vesicles.

**FIGURE 5:**
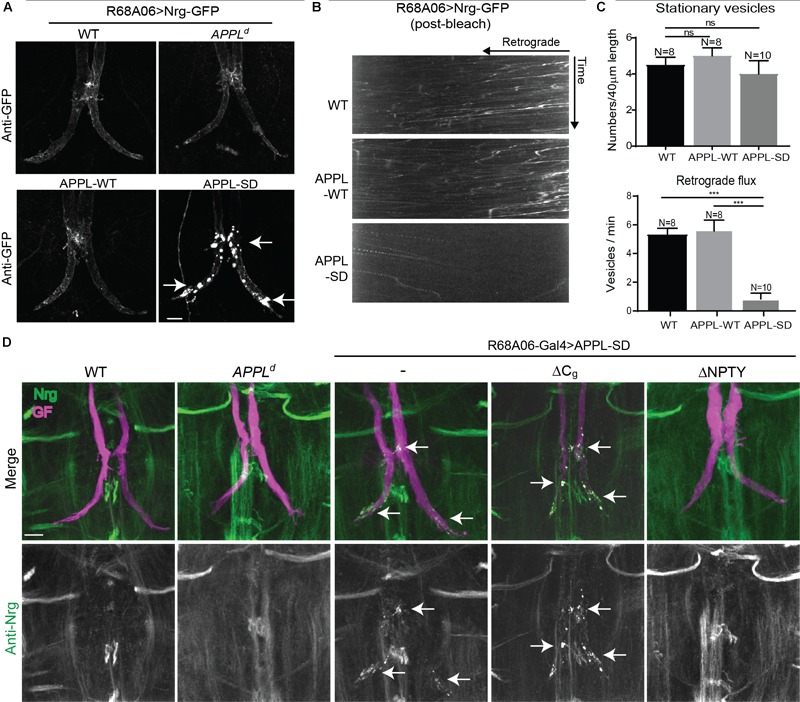
Nrg localization and trafficking in GF terminal of APPL gain and loss of function mutants. **(A)** Nrg-GFP was conditionally expressed in GFs of wildtype and in *APPL*^d^ null mutants with the R68A06 Gal4-line as well as co-expressed with wildtype (APPL-WT) and secretion-deficient (APPL-SD) APPL after GF synapse formation. Nrg accumulations in GF terminals are indicated by arrows. Scale bar represents 10 μm. **(B)** Kymographs of axonal transport of Nrg-GFP vesicles in wildtype (WT) control animals and when co-expressed with APPL-WT or APPL-SD using R68A06 Gal4-line (Supplementary Videos S8–S10). **(C)** Quantification of Nrg-GFP vesicle trafficking in wildtype background as well as when co-expressed with APPL-WT and APPL-SD. Numbers of stationary vesicles, and numbers of vesicles moving in retrograde direction (Flux) were analyzed. N indicates numbers of axons assessed. Error bars represents standard error mean. Statistical significance between genotypes was assessed using Student’s *t*-test (ns, non-significant, *p* > 0.05, ^∗∗∗^*p* < 0.001). **(D)** Localization of endogenous Nrg (green) labeled by an antibody against the intracellular domain of Nrg in wildtype and in *APPL*^d^ null mutants as well as when with APPL-WT, APPL-SD, APPL-SD-ΔCg, and, APPL-SD-ΔNPTY were expressed in GFs with the R68A06 Gal4-line. GFs were labeled by dye injections of rhodamine-dextran (magenta) into the GF axons. Nrg accumulations in GF terminals are indicated by arrows. Scale bar represents 10 μm.

When co-expressed with APPL-SD using R68A06-Gal4, Nrg-GFP accumulated in all GF terminals with no apparent morphological defects in 2-5 days old animals (*n* = 12). However, Nrg-GFP did not accumulate in GF terminals when co-expressed with APPL-WT (*n* = 10) or when expressed in *APPL^d^* null mutants (*n* = 12) or in wildtype (*n* = 10) background (Figure 5A). Similarly, immunolabeling with a monoclonal antibody (BP104) against the intracellular domain of Nrg revealed that the expression of APPL-SD (*n* = 12) but not of APPL-WT (*n* = 16) with either the R68A06 or the GF-Split Gal4-line resulted in accumulation of endogenous Nrg in all dye-injected GFs as well (Figure 5D). The Nrg accumulations were observed in the GF terminals and in the axons around the synaptic contact sites with the PSIs but not in the cervical connective (Figure 5A,D). The extent of accumulation of endogenous Nrg was lower than of Nrg-GFP in all cases, which is likely due to that transgenic expression results in higher protein levels of Nrg-GFP than of endogenous Nrg.

Consistent with the observed accumulation of Nrg in GF terminals, we found that the retrograde flux of Nrg-GFP vesicles in axons imaged in the cervical connective was significantly reduced in APPL-SD expressing but not in APPL-WT expressing animals when compared to controls (Figure 5B,C and Supplementary Videos S8–S10). While expression of both, APPL-WT or APPL-SD, causes accumulation of synaptotagmin along larval motoneuron axons (Torroja et al., 1999a; Gunawardena and Goldstein, 2001), we did not observe an increase of stationary Nrg-GFP vesicles in GF axons in the cervical connective with the co-expression of either construct (Figure 5C and Supplementary Videos S8, S9). This suggests the reduced flux of Nrg-GFP vesicles in GF axons expressing APPL-SD are primarily due to a trafficking defect at the synapse, while the reduced flux in APPL null mutants is likely to be a consequence of a defect during axonal transport as indicated by the increase of Nrg stationary vesicles along the axon.

The finding that Nrg did not selectively accumulate in the GF terminals of *APPL^d^* null mutants suggests that APPL-SD has a toxic effect at GF synapses. To further gain insight into this toxic mechanism, we expressed available APPL-SD constructs in which the endocytic GYENPTY (APPL-SD-ΔNPTY) or the Go-binding motifs (APPL-SD-ΔC_g_) in the intracellular domain were deleted as well. Deletion of either motif, suppressed the ability of APPL-SD to increase boutons at the larval neuromuscular junction (Torroja et al., 1999b; Merdes et al., 2004; Ashley et al., 2005). However, we found that endogenous Nrg accumulated in all GF terminals that expressed APPL-SD-ΔC_g_ (*n* = 12) but not in animals that expressed APPL-SD-ΔNPTY (*n* = 10) with the R68A06 or GF-Split Gal4-line (Figure 5D). Similarly, expression of APPL-SD-ΔC_g_ but not of APPL-SD-ΔNPTY with the R91H05 Gal4-line resulted in GF terminal retraction (data not shown). These results suggest that the lack of GYENPTY directly or indirectly suppresses the disruptive effect of APPL-SD on retrograde receptor trafficking at the synapse.

### Lack of BACE Cleavage Affects Nrg Sorting at the Synapse

**FIGURE 6:**
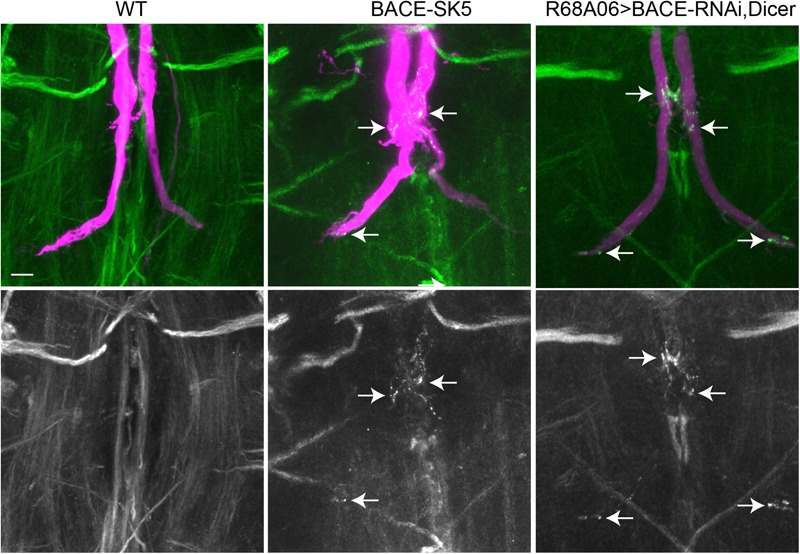
Nrg localization in BACE mutants. Endogenous Nrg was labeled with BP104 antibodies (green) in BACE-SK5 null mutant and in animal co-expression UAS-BACE-RNAi and UAS-Dicer with R68A06-Gal4. The GFs were dye-injected with rhodamine-dextran (magenta). Nrg accumulations in GF terminals are indicated by arrows. Scale bar represents 10 μm.

The finding that Nrg accumulated in vesicular compartment in animals expressing APPL-SD, suggests that β- but not the α-secretase cleavage has a role in sorting of Nrg in endosomal compartments at GF terminals. To test this hypothesis, we assessed localization of endogenous Nrg in GFs of viable BACE mutants (Kondo et al., 2017) as well as in animals in which BACE was knock down post developmentally (Figure 6).

All dye-injected GFs (*n* = 14) of 3–5 days old BACE-SK3 and BACE-SK5 null mutants had GF terminals that did not exhibit any retraction bulbs. However, in the majority (9 out of 14 GFs) of the GFs had Nrg accumulated in their terminals, albeit less extensive than seen with expression of APPL-SD (Figure 6). Similarly, Nrg accumulations can also be observed (2 out of 6 GFs) when a BACE-RNAi construct was co-expressed with Dicer by the R68A06 Gal4-line (Figure 6). No such Nrg accumulations were observed in any of the dye-injected wildtype control animals (*n* = 12). This strongly suggest a role for β-secretase cleavage in regulating Nrg trafficking at GF synapses (Figure 6).

## Discussion

### A Role for APPL in Axonal Transport of Select Vesicles

Numerous studies demonstrate that APPL interaction with motor proteins regulates its own transport (Sabo et al., 1999; Kamal et al., 2001; Matsuda et al., 2001; Scheinfeld et al., 2002; Lazarov et al., 2005; Muresan and Muresan, 2005, 2012; Chiba et al., 2014) but they did not address whether APPL is required for axonal transport of other proteins. Nevertheless, APPL loss of function has been reported to cause axon transport defects of synaptotagmin and CSP (Gunawardena and Goldstein, 2001; Gunawardena et al., 2013). However, it has not been determined if the observed defects are a consequence of general dysfunction in axonal transport, or if APPL has a direct role in axonal transport in anterograde, retrograde or both directions of these proteins. Here, we characterized the effects of APPL loss of function on axonal trafficking of Nrg-GFP and LAMP1-GFP, which do and do not co-localize with APPL vesicles in the GF axons, respectively. In contrast to large synaptotagmin “clogs” in motoneuron axon bundles, we observed in GF axons an increase of individual stationary Nrg-GFP vesicles homogenously along the axons of 1–5-day old *APPL^d^* mutants. However, the fact that APPL loss of function did not exhibit an increase of stationary LAMP1-GFP vesicles or had an effect on their anterograde or retrograde transport, strongly suggests that APPL loss of function does not generally affect dynein or kinesin motor protein functions or the integrity of microtubules. Together with the observation that Nrg-GFP did co-traffic with APPL in GF axons, our results support a direct role for APPL during axonal transport of select vesicles. Ineffectively transported APPL-dependent vesicles are expected to build up over time and large accumulations ultimately are likely to also sterically impact transport of APPL-independent vesicles. Such indirect “traffic jam” effects would also be dependent on axon diameter and numbers of APPL-dependent vesicles transported along the axon. Thus smaller axons, such as larval motoneurons, are potentially more sensitive and therefore may exhibit large “clogs” that impact overall axonal transport more rapidly. Nevertheless, our data implies that the primary cause for the observed trafficking defects in *APPL^d^* mutants is ineffective axonal transport of vesicles it normally traffics in and is not due to altered signaling, which may also non-selectively affect axonal transport.

Despite the strong evidence that APP promotes its own transport in anterograde direction (Sabo et al., 1999; Kamal et al., 2001; Matsuda et al., 2001; Scheinfeld et al., 2002; Lazarov et al., 2005; Muresan and Muresan, 2005, 2012; Chiba et al., 2014), our results suggest that it also has a function during retrograde transport. Nrg co-trafficked with APPL in a retrograde manner and in *APPL^d^* mutants, retrograde but not anterograde flux is reduced. Therefore the increase in Nrg stationary vesicles along the axon is most likely to be the consequence of defects in effectively maintaining transport in the retrograde direction. APP binds JIP1, which does regulate axonal transport of APP bi-directionally (Fu and Holzbaur, 2013; Chiba et al., 2014). While Jun N-terminal kinase (JNK) phosphorylated JIP1 promotes anterograde transport, un-phosphorylated JIP1 promotes retrograde transport. If APP has a role in affecting axonal transport bi-directionally, it could have a regulatory function or serve as an adaptor protein that binds to either antero- or retrograde designated vesicles and thereby promotes directionality of associated proteins. In the first scenario, APP may associate with different proteins that affect the JIP1 phosphorylation status during antero- and retrograde transport. Alternatively, distinctly post-translationally modified APPL isoforms could bind to either phosphorylated or un-phosphorylated JIP1.

The labeling of accumulated vesicles in Lis1-RNAi animals with N-terminal APPL antibodies suggests that either full-length APPL or BACE-cleaved APPL is present in vesicle designated for retrograde transported. The co-localization and co-trafficking experiments (Figure 1C,D), using C-terminally tagged APPL and Nrg, suggests that the C-terminus of APPL is present in APPL-Nrg vesicles as well. However, the optical resolution of confocal microcopy does not allow to distinguish between the presence of only full-length APPL, the presence of full-length as well as N-terminal APPL fragments or the presence of a soluble N-terminal fragment inside the vesicle and a βAPP transmembrane fragment. Calsyntenin-1 has been shown to prevent APP cleavage during anterograde axonal transport (Steuble et al., 2012), and it is conceivable that a yet unidentified protein may prevent presenilin cleavage of an βAPP transmembrane fragment during retrograde transport.

### A Role for Proteolytic Cleavage of APPL in Retrograde Sorting of Cargos at the Synapse

Expression of APPL-SD, but not of APPL-WT resulted in progressive retraction of the GF axons, which is likely to be caused by its disruptive effect on retrograde trafficking of Nrg and other proteins at the synapse. We and others previously showed that inhibition of retrograde transport and altered signaling due to trafficking defects during a critical period results in GF retraction (Allen et al., 1999; Murphey et al., 2003; Godenschwege and Murphey, 2009; Kudumala et al., 2017). While the gain of function effects of APPL-SD and APPL-WT are distinctive from phenotypes observed at the larval neuromuscular junction (Torroja et al., 1999b), they are consistent with phenotypes observed in the *Drosophila* eye (Bolkan et al., 2012). Here, expression of APPL-SD but not of APPL-WT in the retina promoted lamina degeneration, while cleavage of APPL by β-secretase was shown to be essential for glia survival (Bolkan et al., 2012). Our finding that Nrg accumulated at GF synaptic sites in APPL-SD expressing animals, suggests that proteolytic cleavage of APPL has a role in promoting retrograde transport of Nrg from the synapse. However, while stationary Nrg vesicles increased along the GF axons in *APPL^d^* mutants, we did not observe any particular Nrg accumulation at the GF terminals, suggesting that APPL-SD acts as a toxic protein in endosomes at the synapse. APP and BACE-1 were shown to interact at presynaptic sites in hippocampal neurons and BACE-1 cleaves APP in early endosomes after endocytosis, (Rajendran et al., 2006; Rajendran and Annaert, 2012; Das et al., 2016). Therefore, the inability of APPL-SD to bind or be cleaved by *Drosophila* BACE, may affect its own sorting from early to late endosomal compartments and thereby also causing trafficking defects of proteins that are associated with it. Therefore, we propose that full-length APP in endosomal compartments at the synapse has toxic effects on retrograde trafficking if it cannot be proteolytic cleaved.

In support of this hypothesis, we found that Nrg accumulated in BACE null mutants, but the phenotype was less severe than with overexpression of APPL-SD. A likely reason is that in the absence of BACE, endogenous APPL but not APPL-SD is still cleaved by α-secretase Kuzbanian (Kuz), while the amount of APPL present in endosomal compartments is also dependent on the amount of full-length APPL normally endocytosed. In addition, neuronal activity has a role in converging APP and BACE to the same vesicle as well (Das et al., 2013). If during GF synapse formation APPL is primarily cleaved by Kuz, the lack of BACE would only generate little or no full-length APPL in endosomal compartments can interfere with normal retrograde trafficking. Therefore, axon terminal retraction may not occur in BACE mutants, if BACE cleavage only has a role in regulating retrograde APP trafficking in mature synaptic terminals in an activity-dependent manner. In this case, inhibition of α-secretase cleavage in BACE mutants should enhance the retrograde trafficking defects, by promoting the amyloidogenic pathway, which would result in increased levels of full-length APPL in endosomal compartments. Inhibition of presenilin cleavage may further enhance the BACE trafficking defects, if it is able to cleave full-length APPL in endosomal compartments independent of BACE cleavage. A direct role of BACE cleavage in regulating retrograde trafficking at the mature synapse is also suggested by studies of APP mutations that suppress or enhance BACE-1 cleavage, which promotes its own transport in antero or retrograde direction, respectively (Rodrigues et al., 2012). The majority of retrograde and anterograde APP vesicles in axon of cultured hippocampal neurons co-traffic with BACE-1 (Das et al., 2016). It is unknown what the distinction is between antero- and retrograde APP/BACE-1 vesicles and whether APP is already cleaved, is bound to but not cleaved by BACE-1 or not associated with BACE-1 in these vesicles. Presenilin levels affect APP velocities during axonal transport (Gunawardena et al., 2013). Therefore, presenilin association with βAPP/BACE-1 vesicles may regulate retrograde sorting at terminal and/or directionality during axonal transport.

The lack of the endocytic GYENPTY motif suppressed the disruptive effect of APPL-SD (Lai et al., 1998; Perez et al., 1999). Recent studies showed that gene editing of APP, which leads to removal of part of the intracellular domain including the GYENPTY motif, does not affect post-Golgi trafficking (Sun et al., 2019). The impaired endocytosis resulted in increased APP surface levels while attenuating the amyloid pathway but did not cause any noticeable disrupted effect on neuronal physiology. Therefore, such gene-editing is now considered as a potential therapeutic tool to prevent the intracellular generation of toxic Aβ42 peptides (Sun et al., 2019). Mechanistically similar, the prevention of endocytosis of APPL-SD-ΔNPTY may avoid its presence in endosomes and therefore it does not interfere with retrograde trafficking of Nrg. Alternatively, the failure of proteolytic processing of APPL-SD may result in its increase at the surface, if endocytosed full-length APPL that cannot be cleaved by BACE, is recycled back to the surface. This could lead to altered signaling of plasma membrane proteins that interact with APPL, which causes inhibition of retrograde transport. In this scenario, the absence of the GYENPTY motif suppresses this altered signaling. The GYENPTY motif interacts with numerous proteins such as Fe65, X11/Mint as well as JIP1 by which it may regulate or associate with plasma membrane proteins (Matsuda et al., 2001; Scheinfeld et al., 2002; Ashley et al., 2005; Fu and Holzbaur, 2013; van der Kant and Goldstein, 2015).

Expression of APPL-SD did not cause retraction or degeneration of larval motoneurons, albeit like APPL-WT, it causes axonal transport defects (Torroja et al., 1999a; Gunawardena and Goldstein, 2001), suggesting that APPL functions differently in different type of neurons. This could be due to that APPL is predominantly cleaved by α- but not β-secretase in larval motoneurons as discussed above. However, APPL is likely to affect trafficking of transmembrane proteins other than Nrg as well. Thus, it is possible that APPL-SD leads to sorting defects of particular cargoes present in the GF but not in motoneurons, which inhibits endosomal sorting and promotes axonal retraction. APPL processing and trafficking of the full-length protein and fragments is distinctive in different types of neurons (Torroja et al., 1996; Ramaker et al., 2016) and with respect to its functional roles in neurite growth (Singh and Mlodzik, 2012; Mora et al., 2013; Soldano et al., 2013; Singh et al., 2017), synapse formation (Torroja et al., 1999b), and in pruning of GF as well (Figure 4A). Death Receptor 6 has been suggested to regulate axonal pruning via its interaction with vertebrate APP (Nikolaev et al., 2009; Kallop et al., 2014; Olsen et al., 2014; Marik et al., 2016). Therefore, altered signaling at the plasma membrane or defective sorting of a tumor necrosis factor receptor may cause the observed degenerative phenotypes in some but not all cell types of APPL-SD expressing animals.

In summary, our results support a direct role for APPL in the efficacy of retrograde axonal transport of select vesicles suggesting that reduced retrograde signaling of associated cargo protein contributes to loss of function phenotypes, such as pruning, synapse formation, and neurite growth. In addition, we provide evidence that proteolytic cleavage of APPL is essential for proper trafficking of Nrg proteins at the synapse. The finding that APPL gain and loss of function as well as expression of APPL incapable of being cleaved, have distinctive effects in the GFs than in larval motoneurons, while the latter causes degenerative phenotypes in the GFs and in the retina (Torroja et al., 1999b; Bolkan et al., 2012), highlights the importance of studying the endogenous as well as the pathogenic role of APP in distinct types of cells. Compounds developed to treat AD that inhibit APP cleavage or alter its expression levels are likely to have distinct effects on different types of cells.

## Materials and Methods

### Fly Stocks and UAS-APPL-GFP Generation

The following stocks were obtained from the Bloomington Stock Center (Indiana, United States): *w^1118^* (wildtype control, Cat#3605, RRID:BDSC_3605), *Appl^d^* (Cat#43632, RRID:BDSC_43632), UAS-APPL-WT (Cat#38403, RRID:BDSC_38403), UAS-APPL-SD (Cat#29863, RRID:BDSC_29863), UAS-APPL-SD-ΔNPTY (Cat#29864, RRID:BDSC_29864), UAS-APPL-SD-ΔC_g_ (Cat#32041, RRID:BDSC_32041), UAS-APPL-V5 (Cat#63222, RRID:BDSC_63222), UAS-LAMP1-GFP (Cat#42714,RRID:BDSC_42714, rebalanced to drive expression with R68A06 instead of nSyb-Gal4), UAS-CD8-GFP (Cat#5137, RRID:BDSC_5137), R91H05-Gal4 (Cat#40594, RRID:BDSC_40594), R68A06-Gal4, (Cat#39449, RRID:BDSC_39449), UAS-Lis1-RNAi (Cat#35043, RRID:BDSC_35043), and UAS-Dicer (Cat#24659, RRID:BDSC_24650). UAS-BACE-RNAi (Cat#v15541) was obtained from the Vienna Drosophila Resource Center. UAS-Nrg-GFP and UAS-Nrg-mCherry were obtained from the Jan Pielage lab and the trafficking of the constructs in the GFs has been previously characterized (Enneking et al., 2013; Siegenthaler et al., 2015; Kudumala et al., 2017). GF-Split Gal4-line (Cat#79603, RRID:BDSC_79602) was obtained from the Gwyneth Card lab (von Reyn et al., 2014). Drosophila BACE null mutants (SK3 and SK5) were obtain from Kondo et al. (2017).

UAS-APPL-GFP was generated by cloning the APPL cDNA (obtained from the Drosophila Genomics Resource Center, DGRC, clone GH04413, Cat#5877) into the pENTR^TM^ vector (Cat#A10467, Invitrogen) and subsequently recombined into the pTWG vector (Cat#1076, DGRC) using the Invitrogen Gateway^TM^ Cloning System. All cloning products were confirmed by Sanger sequencing (GeneWiz, Inc.). Transgenic UAS-APPL-GFP lines were established on the second and third chromosomes (BestGene, Inc.).

R91H05-Gal4 was used to drive cell-autonomous expression of UAS-constructs throughout GF development, whereas R68A06-Gal4 and the GF-Split Gal4-line were used to drive expression after GF terminal development (Pfeiffer et al., 2008; Lee and Godenschwege, 2015; Kudumala et al., 2017). Flies were reared at 25°C on standard fly media and 2–5-day-old flies were used in all experiments unless otherwise specified.

### Live-Imaging and Trafficking Analysis

Live imaging was performed similar as previously described (Kudumala et al., 2017). Isolated central nervous systems of 3–5-day old animals were mounted onto Poly-L-Lysine (Sigma Aldrich, P0879) coated glass bottom dishes containing saline (NaCl 128 mM, KCl 2 mM, CaCl_2_ 1.8 mM, MgCl_2_ 4 mM, HEPES 5 mM, sucrose 35.5 mM, pH = 7.2) (FluoroDish FD35, World Precision Instruments). The GF axons were imaged at the dorsal side in the cervical connective with an upright Nikon A1 or an inverted Nikon A1 plus Confocal with GaAsP multi-detector units using a CFl Plan 100 × /1.1 NA water immersion lens or a CFI Plan APO lamba 60 × /NA1.4 oil objective at room temperature (20–23°C). Live-recordings were obtained at 1–2 frames/second, 512 or 1024 resolution, 2–10% of 488 nm and/or 561 nm excitation laser power, and with a 90% open pinhole for up to 10 min. When necessary, photo-bleaching was performed at 40–50% excitation laser power for 0.5–1 min. Nikon Elements Advanced Research 4.4 was used for video recordings, kymograph generation, and analysis. Flux (vesicles/min) was determined as the number of vesicles passing through an arbitrary vertical line along the GF axon per minute. Stationary vesicles were defined as vesicles that remain within their initial position ± 0.1 μm for at least 1 min of recording. The average number of stationary vesicles per 40 μm length of unbleached axons were determined. Statistical significance between groups was calculated using Student’s *t*-test or one-way ANOVA followed by Tukey’s *post hoc* test using GraphPad’s Prism 7.03 (RRID:SCR_010279). Data are shown as the mean ± standard error of the mean (SEM) where number of asterisks indicate degree of significance (^∗^*p* ≤ 0.05, ^∗∗^*p* ≤ 0.01, ^∗∗∗^*p* ≤ 0.001).

### Dye-Injection and Immunohistochemistry

Giant Fibers were labeled by injections of tetramethylrhodamine isothiocyanate-Dextran (Cat# 42874 Sigma) into the GF axons or by expression of membrane-bound GFP (UAS-CD8-GFP). The procedures for adult *Drosophila* nervous system dissection, dye-injection, and immunolabeling have been previously described in detail (Boerner and Godenschwege, 2010a,b). In brief, dissected nervous systems were fixed (4% paraformaldehyde), permeabilized (0.5% Triton X-100 in 1 × phosphate buffered saline), and incubated with the following primary antibody: rabbit anti-APP-extracellular (1:500, Santa Cruz Biotechnology Cat# sc-98268, RRID:AB_1563367), chicken anti-GFP (1:1,000, Abcam Cat# ab13970, RRID:AB_300798 or 1:500, Molecular Probes Cat# A-11122, RRID:AB_221569), mouse anti-V5 (1:500, Thermo Fisher Scientific Cat# R960CUS, RRID:AB_2792973), and anti-Neuroglian (1:10, DSHB, Cat#BP104, RRID:AB_528402). The following secondary antibodies were used: anti-Rabbit Cy3 (1:500, Jackson ImmunoResearch Labs Cat# 111-165-003, RRID:AB_2338000), anti-Chicken DyLight 488 (1:1,000, Thermo Fisher Scientific Cat# SA5-10070, RRID:AB_2556650), anti-mouse Cy3 (1:750, Jackson ImmunoResearch Labs Cat# 115-165-020, RRID:AB_2338683), and anti-mouse DyLight 649 (1:500, Jackson ImmunoResearch Labs Cat# 115-495-075, RRID:AB_2338809). Samples were scanned at a resolution of 1024 × 1024 pixels, 2.4-pixel dwell, and 0.2 μm step size with a Nikon A1R plus confocal microscope using a 60 × /1.4 NA oil immersion objective lens. Images were processed using Nikon Elements Advance Research 4.4 (RRID:SCR_014329) and Adobe Illustrator (RRID:SCR_010279) software. For images in Figure 1B the Binary Editor and the ND Images Arithmetic function of Nikon Elements Advanced Research 4.4 software were used to trace, 3D reconstruct the GFs, and extract anti-APPL labeling that localizes to the 3D reconstructed GFs.

### Giant Fiber Electrophysiology

Electrophysiological recordings were obtained as previously described in detail (Allen and Godenschwege, 2010). To determine the presence and reliability (Following frequency) of the GF to TTMn synapse, 10 trains of 10 stimuli were given at 100 Hz with an interval of 2 s between the trains. The percent of the total responses was calculated. All of the traces were recorded, stored and analyzed using pClamp 10.3 (Molecular Devices, RRID:SCR_011323) software. Statistical significance between genotypes was calculated using Student’s *t*-test or one-way ANOVA followed by Tukey’s *post hoc* test using GraphPad’s Prism 7.03 (RRID:SCR_002798). Data are shown as the mean ± SEM where number of asterisks indicate degree of significance (^∗^*p* ≤ 0.05, ^∗∗^*p* ≤ 0.01, ^∗∗∗^*p* ≤ 0.001).

## Data Availability

The raw data supporting the conclusions of this manuscript will be made available by the authors, without undue reservation, to any qualified researcher.

## Author Contributions

TP conducted the experiments, prepared the figures, analyzed and interpreted the data, and wrote and edited the manuscript. SK conducted the experiments, prepared the figures, analyzed the data, edited the manuscript. RP conducted the experiments, analyzed the data. TG conceived, designed, planned, and supervised the entire study, secured the funding, interpreted the data, prepared the figures, wrote and edited the manuscript.

## Conflict of Interest Statement

The authors declare that the research was conducted in the absence of any commercial or financial relationships that could be construed as a potential conflict of interest.
